# Cost-effectiveness of consolidation durvalumab for inoperable stage III non-small cell lung cancer in Vietnam

**DOI:** 10.1136/bmjopen-2024-083895

**Published:** 2024-08-30

**Authors:** Vu Quynh Mai, Lars Lindholm, Hoang Van Minh, Sun Sun, Kim Bao Giang, Klas-Göran Sahlén

**Affiliations:** 1 Epidemiology and Global Public Health, Umea Universitet, Umea, Sweden; 2 Hanoi University of Public Health, Hanoi, Viet Nam; 3 Umea Universitet, Umea, Sweden; 4 Department of Epidemiology and Global Health, Umeå University, Umea, Sweden; 5 Department of Learning, Informatics Management and Ethics Karolinska Institute, Sweden; 6 Preventive Medicine and Public Health, Hanoi Medical University, Hanoi, Viet Nam; 7 Public Health and Clinical Medicine, Umea University, Umea, Sweden

**Keywords:** health economics, lung diseases, chemotherapy

## Abstract

**Abstract:**

**Background:**

This study aimed to assess the cost-effectiveness of durvalumab as a treatment option for patients with inoperable stage III non-small cell lung cancer (NSCLC) from healthcare and partial societal perspectives in Vietnam.

**Method:**

A lifetime partitioned survival model was used to evaluate the costs and quality-adjusted life years (QALYs) associated with consolidation durvalumab in comparison with the standard of care alone. Local costs and utilities were incorporated into the model. In the base-case analysis, no discount was applied to the acquisition cost of durvalumab. Scenario-based, one-way and probabilistic-sensitivity analyses were conducted.

**Results:**

The base-case analysis revealed that the intervention resulted in an increase of 1.38 life years or 1.08 QALYs for patients, but the intervention was not deemed cost-effective from either perspective in the base-case analysis. However, with a 70% reduction in the durvalumab acquisition cost, the intervention was observed to be cost-effective when evaluated from a healthcare perspective and when examining the undiscounted results from a partial societal standpoint.

**Conclusion:**

This study provides evidence regarding the cost-effectiveness of durvalumab for the treatment of inoperable stage III NSCLC in Vietnam for various scenarios. The intervention was not cost-effective at full acquisition cost, but it is important to acknowledge that cost-effectiveness arguments alone cannot solely guide decision-makers in Vietnam; other criteria, such as budget impact and ethical concerns, are crucial factors to consider in decision-making processes.

**STRENGTHS AND LIMITATIONS OF THIS STUDY**

- This study employed the most updated 5 year survival rate data of durvalumab treatment for inoperable stage III non-small cell lung cancer (NSCLC) to reconstruct survival inputs for our cost-effectiveness analysis.
- Local sources were employed to estimate the costs and utilities associated with the treatment for inoperable stage III NSCLC, offering insights for Vietnamese decision-makers.
- The clinical inputs were derived from the PACIFIC trial, which may raise concerns about their representativeness for Vietnamese inoperable stage III NSCLC patients.

## Introduction

 Lung cancer is a significant contributor to cancer-related mortality in Vietnam, ranking as the second leading cause of death.^1^ In 2020, the mortality rates for lung cancer were reported as 35.1 per 100 000 among males and 13.8 per 100 000 among females.^1^ Non-small cell lung cancer (NSCLC) represents the majority (approximately 84%) of lung cancer cases. The Vietnamese Ministry of Health issued treatment guidelines specifically targeting NSCLC in 2018 (online supplemental figure 1).^2^ According to these guidelines, surgical resection is often recommended for stage III NSCLC patients; however, approximately 30% are deemed ineligible for the operation due to their poor health status or tumour location.^3^ Patients with inoperable stage III NSCLC are typically managed through concurrent or sequential chemoradiotherapy.^2^ However, evidence indicates that the 5 year overall survival rate for these patients is unfortunately below 25%.^4^ In recent years, consolidation immunotherapy has shown promise in extending the survival of patients with inoperable stage III NSCLC, and durvalumab is one example of such an immunotherapy.^5^

A randomised controlled trial called PACIFIC investigated the efficacy of durvalumab consolidation in patients with inoperable stage III NSCLC who had not experienced disease progression following two or more cycles of platinum-based chemoradiotherapy.^5^ The trial demonstrated encouraging outcomes, including a 42.9% probability of survival at 75 months (stratified HR to placebo: 0.72) and a 33.1% probability of progression-free survival at 72 months (stratified HR to placebo: 0.55).^5^ Working as an active ingredient in Imfinzi, durvalumab is an immune-checkpoint inhibitor that functions as an anti-programmed cell-death ligand 1 (PD-L1) antibody.^6^ However, it is important to note that durvalumab can also lead to adverse events, such as cough, fatigue, dyspnoea, pneumonitis, pneumonia and anaemia.^7^ According to the pharmaceutical provider, durvalumab is administered as a monotherapy when the cancer cells have not metastasised beyond the chest, are unresectable and have shown a response or stabilisation following initial chemotherapy containing platinum that was administered concurrently with radiation therapy in the treatment of inoperable stage III NSCLC. The recommended duration of durvalumab therapy is a maximum of 12 months or until disease progression or unacceptable toxicity occurs, with the dosage based on the patient’s body weight. Although durvalumab is suggested in the Vietnamese treatment guidelines for inoperable stage III NSCLC (online supplemental figure 1),^2^ it is not covered by national health insurance in Vietnam.

Vietnam has made significant strides towards universal health coverage (UHC), including the implementation of compulsory social health insurance (SHI) that covers all Vietnamese citizens.^8^ The SHI package encompasses provisions as detailed in the Vietnamese essential drug list and the list of reimbursed medical services.^9 10^ The SHI has a single national fund that reimburses the cost of healthcare services provided by both public and private providers^8^; the Ministry of Health establishes reimbursement levels and rates for healthcare services covered by the SHI on an annual basis.^10^ Reimbursements for SHI drugs are determined based on purchasing prices derived from national, centralised drug procurement and the SHI reimbursement rates for drugs.^9^ Durvalumab is currently not included in the SHI scheme, meaning that patients hoping to extend their lives with this immunotherapy must bear the entire cost themselves, causing a substantial financial burden for them and their families. This is exacerbated by the more general fact that healthcare expenditure has pushed approximately 37.4% of cancer patients in Vietnam into poverty.^11^

Three dimensions of UHC can be identified: health insurance coverage, provision of essential healthcare services and financial protection for patients.^12^ To achieve UHC, Vietnam not only needs to expand the coverage of its SHI but also enhance the SHI package and reimbursement system. The country has initiated plans to annually update the SHI package and reimbursement criteria, meaning that the inclusion of any new drug to the SHI must undergo a health technology assessment process, which requires evidence regarding effectiveness, safety and cost-effectiveness.^13^ Global evidence from studies conducted in countries such as the USA, Switzerland and China has demonstrated the cost-effectiveness of durvalumab as a treatment for inoperable stage III NSCLC,^14^,^18^ although a study in Italy has cast doubt on this.^19^ These studies used survival and safety data from the PACIFIC trial for their cost-effectiveness analyses, adjusting for patient characteristics, costs and utility based on local statistics.^14^,^1719^ While evidence on the NSCLC population in Vietnam has been briefly mentioned elsewhere,^20^ there is a lack of evidence regarding the cost-effectiveness of durvalumab for inoperable stage III NSCLC in Vietnam. Therefore, this study was conducted to provide local evidence on the economic aspect of this intervention. The objective of the study was to explore the cost-effectiveness of durvalumab in treating inoperable stage III NSCLC in Vietnam from healthcare and partial societal perspectives.

## Method

A cost-utility analysis model was developed using an Excel spreadsheet. In Vietnam, the standard of care for inoperable stage III NSCLC involves chemo-radiotherapy without consolidation immunotherapy.^2^ Therefore, the evaluated intervention was the standard of care combined with durvalumab consolidation, while the comparator was the standard of care. All cost data is presented in Vietnamese Dong (VND), adjusted to 2021 prices using the Vietnamese commercial price indices.

### Study population

The study focused on patients who were eligible for durvalumab in Vietnam, had inoperable stage III NSCLC and had not shown disease progression after two or more cycles of chemotherapy and radiotherapy (online supplemental figure 1). The eligible patients received the intervention for a maximum duration of 12 months. We used published data indicating that the average age of NSCLC patients was 59.96 years and 59% of patients are male (table 1)^20^ to produce the model. Due to the lack of information on the average weights of patients, we assumed that NSCLC patients would have a similar weight to the general population, as was reported in 2015.^21^ The body surface area was estimated based on the average weight and height of the Vietnamese population.^22^

**Table 1:** Parameters used in the cost-effectiveness model

Parameter	Base-case value	SE	Lower bound	Upper bound	Distribution	Source
Model setting						
Cost discounting rate	0.03	0.00*	0.00	0.05	Beta	
Effectiveness discounting rate	0.03	0.00*	0.00	0.05	Beta	
Time horizon (year)	15.00	1.50*	10.00	20.00	Normal	
Patient age (year)	59.80	5.98*	58.67	60.49	Normal	^20^
Patient body surface area (m^2^)	1.61	0.18	1.25	1.96	Normal	
Patient weight (kg)	56.96	0.67	55.65	58.28	Normal	^21^
Percentage of male patients	0.59	0.06*	0.47	0.70	Beta	^20^
PFS HR	0.52	0.06	0.45	0.68	Beta	^5^
OS HR	0.72	0.08	0.59	0.89	Beta	^5^
Durvalumab: rate of having pneumonia	0.04	0.004*	0.04	0.04	Beta	^7^
Durvalumab: rate of having pneumonitis	0.03	0.003*	0.02	0.02	Beta	^7^
Durvalumab: rate of having anaemia	0.02	0.002*	0.02	0.02	Beta	^7^
Durvalumab: rate of having dyspnoea	0.02	0.002*	0.01	0.01	Beta	^7^
Standard of care: rate of having pneumonia	0.04	0.004*	0.03	0.03	Beta	^7^
Standard of care: rate of having pneumonitis	0.03	0.003*	0.03	0.03	Beta	^7^
Standard of care: rate of having anaemia	0.02	0.002*	0.01	0.01	Beta	^7^
Standard of care: rate of having dyspnoea	0.03	0.003*	0.02	0.02	Beta	^7^
Utility						
Utility at PF	0.76	(0.05)	0.66	0.85	Beta	^20^
Utility at PD	0.68	(0.04)	0.61	0.75	Beta	^20^
Disutility pneumonia	(0.07)	0.02	(0.11)	(0.04)	Beta	^31^
Disutility anaemia	(0.07)	0.02	(0.11)	(0.04)	Beta	^29^
Disutility pneumonitis	(0.07)	0.02	(0.11)	(0.04)	Beta	^30^
Disutility dyspnoea	(0.05)	0.01	(0.08)	(0.03)	Beta	^30^
Micro costing direct medical cost from the SHI reimbursement (million VND)						
Monitoring cost per administration	405.20	40.52	405.20	405.20	Gamma	
Durvalumab acquisition cost per course	48 695.02	1.74	36 067.91	61 322.13	Gamma	
Administration cost per course	111.42	11.14	109.75	113.09	Gamma	
Maximum subsequence cost	66 795.14	6679.51	66 391.16	67 199.12	Gamma	
AE- Anaemia treatment/course	6249.52	624.95	5742.35	6756.69	Gamma	
AE- Dyspnoea treatment/course	1512.35	151.24	1512.35	1512.35	Gamma	
AE- Pneumonia treatment/course	9211.92	921.19	9210.47	9213.37	Gamma	
AE- Pneumonitis treatment/course	9508.60	950.86	9399.55	9617.65	Gamma	
Out-of-pocket payments (thousand VND)						
Direct medical cost at PF	11 817.04	(3,394.57)	5080.63	18 553.45	Gamma	^20^
Direct medical cost at PD	12 286.77	(2,953.42)	6425.80	18 147.74	Gamma	^20^
Direct non-medical cost at PF	1841.38	184.14	925.41	2757.35	Gamma	^20^
Direct non-medical cost at PD	2238.99	223.90	925.57	3552.41	Gamma	^20^

* Data presented the estimated SE at 10% of the base-case value.

AEadverse eventOS HRoverall survival HRPDprogressed diseasePFprogression-freePFS HRprogression-free survival HR

### Model setting

A partitioned survival model was employed, consisting of three main stages: progression-free (PF), progressed disease (PD) and death (online supplemental figure 2). The final progression-free survival (PFS) rate, denoted as P1, was calculated as the probability of the PF stage. The probability of death (P3) was estimated as one minus the overall survival (OS) rate, while the probability of PD (P2) was calculated as one minus P1 minus P3. The incremental cost-effectiveness ratio (ICER) (as calculated by ∆cost/∆outcome) and net money benefit (NMB) (as calculated by ∆outcome * threshold - ∆cost) between the intervention and standard of care were estimated. A 15 year time horizon was selected, considering the life expectancy of Vietnamese patients is approximately 75 years^23^ and the average age of NSCLC patients is 59.96.^20^ Costs and outcomes were discounted at a rate of 3% over time. The cost-effectiveness threshold used in the model was 258 million VND, based on the three times gross domestic product (GDP) threshold suggested by the WHO for cost-effectiveness analyses.^24 25^

### Survival data

We employed a method proposed by Wei and Royston^26^ *et al* to reconstruct OS and PFS data based on published evidence from the PACIFIC trial regarding the 5 year survival rate of durvalumab treatment for inoperable stage III NSCLC.^5^ Parametric survival models were used to extrapolate the survival data over time (in months). The selection of the best-fit model was based on information criteria, including the Akaike and Bayesian information criteria. Online supplemental file provides detailed information on the extrapolation process and selection of the best-fit model. The extrapolated survival data, obtained from the best-fit model, was adjusted to ensure it did not surpass the age-sex survival rates suggested in the Vietnamese life table.^23^ For observation periods beyond the specified time points (75 months for OS and 72 months for PFS), the survival data in the durvalumab arm was estimated using HRs and extrapolated survival data from the standard of care arm. Furthermore, adjustments were made to the PFS over time to prevent it from exceeding the corresponding OS (online supplemental figure 3).

### Adverse events

The incidence and severity of adverse events associated with durvalumab in inoperable stage III NSCLC were based on data collected over a 24 month observation period.^7^ It was reported that more than 20% of patients receiving durvalumab experienced adverse events such as cough, fatigue, dyspnoea and pneumonitis.^7^ Considering the potential cost implications and impact on quality of life associated with adverse events, our analysis focused specifically on severe adverse events of Grade three or higher, including pneumonia, pneumonitis, anaemia and dyspnoea (table 1). This approach adheres to the recommendation outlined in the Professional Society for Health Economics and Outcomes Research good practice guidelines for estimating adverse events in economic models.^27^ The definition of ‘severe adverse events’ is previously established and documented.^7^

### Utility

The utility of Vietnamese NSCLC patients was previously reported by Ha, Hoang^20^ in 2018, where a mean utility of 0.66 was derived using the EQ-5D-5L instrument and a pre-published Vietnamese value set. In the present study, the lead author conducted additional analysis on patient utility at different disease stages using the anonymised dataset provided from Ha, Hoang’s^20^ study. This analysis involved data from 400 NSCLC adult patients at six oncology hospitals across northern, central and southern Vietnam.^20^ The health utility values were re-calculated using the recently published Vietnam EQ-5D-5L value set.^28^ The mean utility for NSCLC patients in stage IIIB was found to be 0.76, while for those in stage IV, it was 0.68 (table 1). Due to data limitations, we assumed equivalent mean utilities for PF and stage IIIB patients and for PD and stage IV patients (table 1). Disutility values resulting from severe adverse events were obtained from relevant literature sources.^29^,^31^

### Cost

The costs were estimated from a healthcare perspective and a partial societal perspective.

#### Perspectives

The intention of this analysis was to facilitate the allocation of SHI funds, which necessitated adopting a healthcare perspective. The cost estimation from a healthcare standpoint encompassed the reimbursements provided by the SHI for drugs and medical services. In addition, a partial estimation of societal costs was conducted in this study, incorporating those borne by the SHI fund and patients’ out-of-pocket payments.

#### SHI reimbursements

The health insurance reimbursements encompassed the direct costs of drugs and medical services covered by the SHI. To estimate the costs of drugs and services for inoperable stage III NSCLC in Vietnam, we employed a guideline-based approach. The costing study included the following cost components: (1) acquisition cost of durvalumab, (2) administration cost of durvalumab, (3) disease-monitoring cost, (4) cost of subsequent treatments for patients who experienced disease progression and (5) cost of treating adverse events. The cost of medical services was estimated using the reimbursement prices and rates set by the SHI.^9 10 32^ These reimbursement prices consider necessary resources such as personnel, operations, maintenance and supplies.^10^ The cost of drugs was estimated using the SHI reimbursement rates for drugs, along with average prices obtained from Vietnamese centralised drug procurement.^9 10 32^ For the acquisition cost of durvalumab (Component 1), we used an average of two registration prices: 41 870 745 VND for a box of 500 mg and 10 467 686 VND for a box of 120 mg. We considered a dosage of 10 mg/kg for a maximum of 12 months or until disease progression. The cost of durvalumab administration (Component 2) included expenses such as hospital beds, intravenous infusion and NaCl 0.9 solution. For other cost components, we relied on secondary data collected during the previous work to determine the costs associated with NSCLC. In an earlier study, the lead author conducted interviews with six oncology experts from three national oncology hospitals in the northern, central and southern regions of Vietnam. These interviews aimed to identify common monitoring practices, subsequent treatments and adverse-event management for patients receiving immunotherapy in Vietnam. We aggregated and verified the frequency of prescriptions and average consumption of relevant services per treatment course based on expert consultations and national guidelines on NSCLC treatment.^2^ The disease-monitoring cost (Component 3) covered expenses for health-monitoring services, including periodic check-ups, CT scans and laboratory tests. The subsequent treatments (Component 4) referred to non-immunotherapies. The cost of adverse-event treatments (Component 5) consisted of the expenses associated with managing four severe adverse events: pneumonia, pneumonitis, anaemia and dyspnoea. A detailed description of the guideline-based costing is stated in online supplemental file.

#### Patients’ out-of-pocket expenditure

Our analysis focused on out-of-pocket expenditure incurred by patients in relation to direct medical costs (eg, tests, drugs) and direct non-medical costs (eg, travel, accommodation). Data on these expenses were sourced from the study conducted by Ha, Hoang^20^ in 2018, which surveyed patients about their 12 month out-of-pocket costs during NSCLC treatment, including targeted treatment, monitoring, adverse-event management and non-medical expenses. We analysed the out-of-pocket expenditure at two disease stages (IIIB and IV). Due to limited data, we assumed IIIB expenses mirrored those in the PF stage and IV in the PD stage (table 1).

### Base-case analysis

The base-case analysis was conducted based on several key assumptions: first, all patients were assumed to enter the model at the PF stage and progress forwards accordingly (from PF to PD or death and from PD to death). Second, it was assumed that the durvalumab acquisition cost was estimated at the registration price. Although the actual cost of durvalumab acquisition can vary due to factors such as vial-sharing strategies, discount campaigns and agreements between the pharmaceutical company and the Vietnamese government, the base-case analysis maintained a neutral position by assuming no decrement in durvalumab acquisition cost. The ICER was presented in terms of both incremental cost per LYs gained and incremental cost per QALYs gained. Both discounted and undiscounted results are presented. A detailed model of the base-case analysis and sensitivity analysis is presented in online supplemental file.

### Sensitivity analysis

We conducted a scenario sensitivity analysis, a one-way sensitivity analysis and a probability sensitivity analysis. The scenario sensitivity analysis explored how reducing durvalumab’s acquisition cost by 50% and 70% impacted cost-effectiveness as compared with the base-case analysis, which allowed us to examine different pricing scenarios. In the one-way sensitivity analysis, we varied input parameters within their 95% CI bounds based on their distribution and assumed a 10% SE when bounds were unavailable (table 1).^33^ Tornado charts were used to illustrate the ten most impactful parameters on NMB. For the probabilistic sensitivity analysis, we ran 1000 iterations to address parameter uncertainty and used scatter plots to visualise ICER variation. Cost-effectiveness acceptability curves depicted the average probabilities of the NMB favouring durvalumab, considering different cost-effectiveness thresholds.

### Patient and public involvement

None.

## Results

### Base-case analysis

The base-case analysis showed that the use of durvalumab consolidation was associated with an estimated gain of 1.38 LYs or 1.08 QALYs (table 2). The discounted ICER was estimated to be 683.02 million VND per LY gained and 869.49 million VND per QALY gained from a partial societal perspective and 651.19 million VND per LY gained and 828.97 million VND per QALY gained from a healthcare perspective (table 2). NMB was calculated using the incremental cost and incremental utility, with a GDP-based threshold. The intervention with durvalumab was not considered to be a cost-effective intervention in the base-case analysis from both perspectives (table 2).

**Table 2:** Cost-effectiveness base-case analysis of durvalumab for inoperable stage III NSCLC patients

	Partial societal perspective				Healthcare perspective			
	Consolidation durvalumab	Standard of care	Difference (discounted)	Difference (undiscounted)	Consolidation durvalumab	Standard of care	Difference (discounted)	Difference (undiscounted)
Life Years - LY								
Progression free	4.19	2.28	1.91	2.34	4.19	2.28	1.91	2.34
Progressed disease	1.11	1.64	(0.53)	(0.64)	1.11	1.64	(0.53)	(0.64)
Total LY	**5.30**	**3.92**	**1.38**	**1.71**	**5.30**	**3.92**	**1.38**	**1.71**
Quality-adjusted life years (QALY)								
Progression-free	3.17	1.72	1.45	1.77	3.17	1.72	1.45	1.77
Progressed disease	0.76	1.12	(0.36)	(0.44)	0.76	1.12	(0.36)	(0.44)
Adverse events disutility	(0.00)	(0.00)	(0.00)	(0.00)	(0.00)	(0.00)	(0.00)	(0.00)
Total QALY	**3.93**	**2.84**	**1.08**	**1.34**	**3.93**	**2.84**	**1.08**	**1.34**
Cost (million VND)								
Acquisition and administration	893.07	-	893.07	905.32	893.07	-	893.07	905.32
Monitoring	25.47	18.88	6.59	8.10	25.47	18.88	6.59	8.10
Subsequent therapy	1.51	2.23	(0.73)	(0.87)	1.51	2.23	(0.73)	(0.87)
Adverse events	0.64	0.55	0.09	0.10	0.64	0.55	0.09	0.10
Out-of-pocket: other medical cost	101.00	54.88	46.12	56.50	-	-	-	-
Out-of-pocket: nonmedical cost	4.52	6.70	(2.17)	(2.60)	-	-	-	-
Total cost	**1026.22**	**83.25**	**942.98**	**966.55**	**920.70**	**21.66**	**899.03**	**912.65**

Incremental cost-effectiveness ratio	Discounted value		Undiscounted value		Discounted value		Undiscounted value	
	∆cost/∆LYs	∆cost/∆QALYs	∆cost/∆LYs	∆cost/∆QALYs	∆cost/∆LYs	∆cost/∆QALYs	∆cost/∆LYs	∆cost/∆QALYs
Base-case analysis	683.02	869.49	566.62	722.12	651.19	828.97	535.03	681.85
Durvalumab acquisition cost cut by 50%	360.32	458.69	301.86	384.70	328.49	418.17	270.27	344.44
Durvalumab acquisition cost cut by 70%	231.24	294.38	195.96	249.74	199.41	253.85	164.36	209.47

Net money benefit	Discounted value	Undiscounted value	Discounted value	Undiscounted value
Base-case analysis	(662.90)	(620.89)	(618.96)	(566.99)
Durvalumab acquisition cost cut by 50%	(217.39)	(169.26)	(173.44)	(115.36)
Durvalumab acquisition cost cut by 70%	(39.18)	**11.39**	**4.77**	**65.29**

Notes: Cost results are presented in million VND. Base-case results were estimated based on the following assumptions: durvalumab acquisition cost was estimated without any cost decrement; cost and disutility-related adverse events were included in the analysis; the discounted results were estimated with a discounting rate of 3% to both cost and effectiveness outcomes in 15 years.

### Scenario sensitivity analysis

The scenario sensitivity analysis included two scenarios to assess NMB: (1) intervention with a 50% reduction in durvalumab acquisition cost and (2) intervention with a 70% reduction in acquisition cost (table 2). A 50% reduction in the durvalumab acquisition cost did not cause the consolidation durvalumab intervention to be cost-effective (table 2); however, with a 70% reduction in the durvalumab acquisition cost the intervention was observed to be cost-effective from a healthcare perspective and when examining the undiscounted results from a partial societal perspective (table 2).

### One-way sensitivity analysis

**Figure 1:**
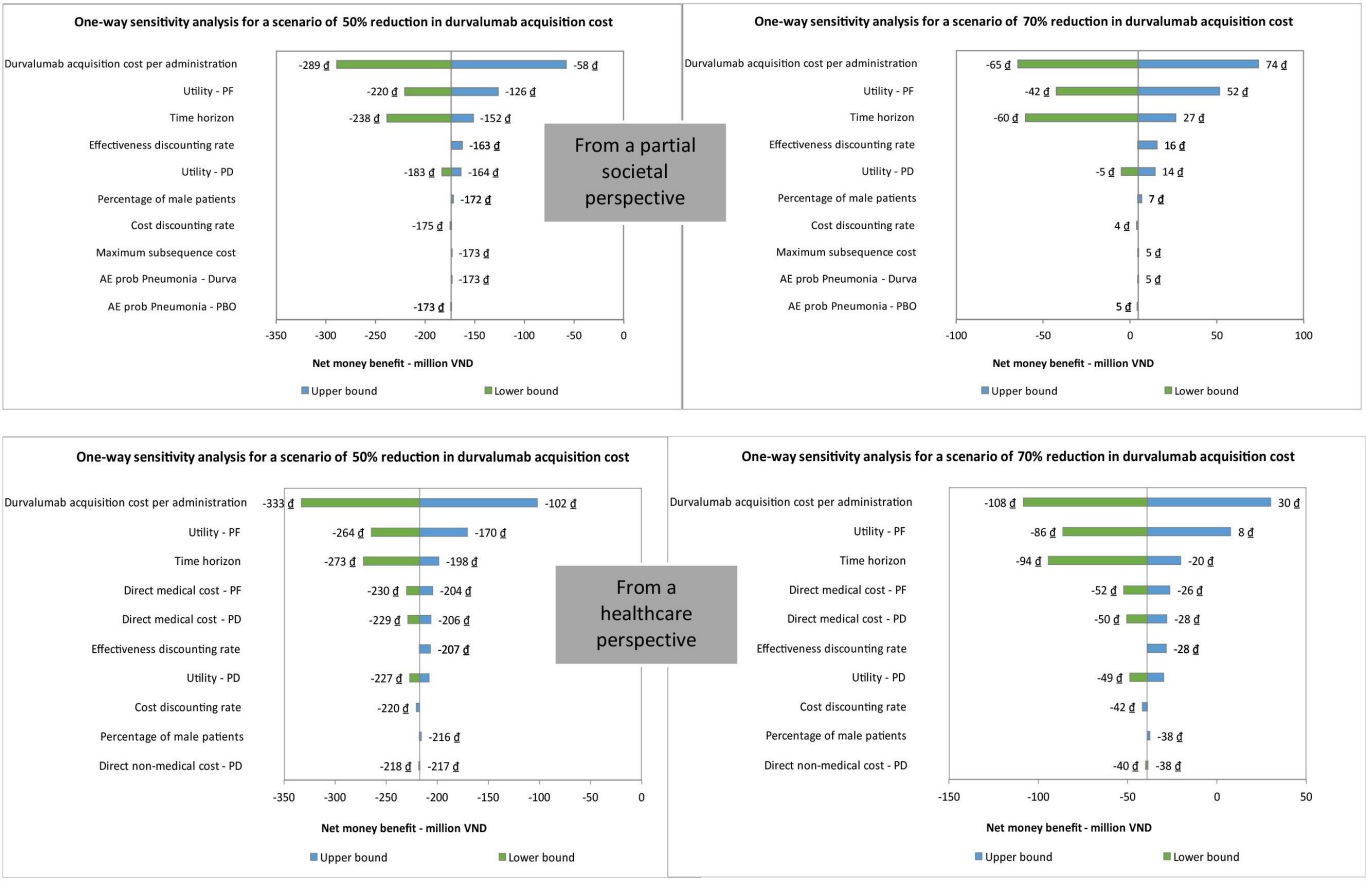
Top ten impact factors on net money benefit in scenarios with reduced durvalumab acquisition cost.

A one-way sensitivity analysis assessed the factors impacting NMB when the durvalumab acquisition cost was reduced by 50% or 70% from both the healthcare and partial societal perspectives. With a 50% cost reduction, no input affected the cost-effectiveness conclusion. In the 70% cost-reduction scenario, changes in durvalumab cost and utility at the PF stage influenced the conclusion from a partial societal perspective (figure 1). From a healthcare perspective, the influential factors included durvalumab cost, utility at both the PF and PD stages, time horizon and discounting rate for QALYs (figure 1).

### Probability sensitivity analysis

**Figure 2:**
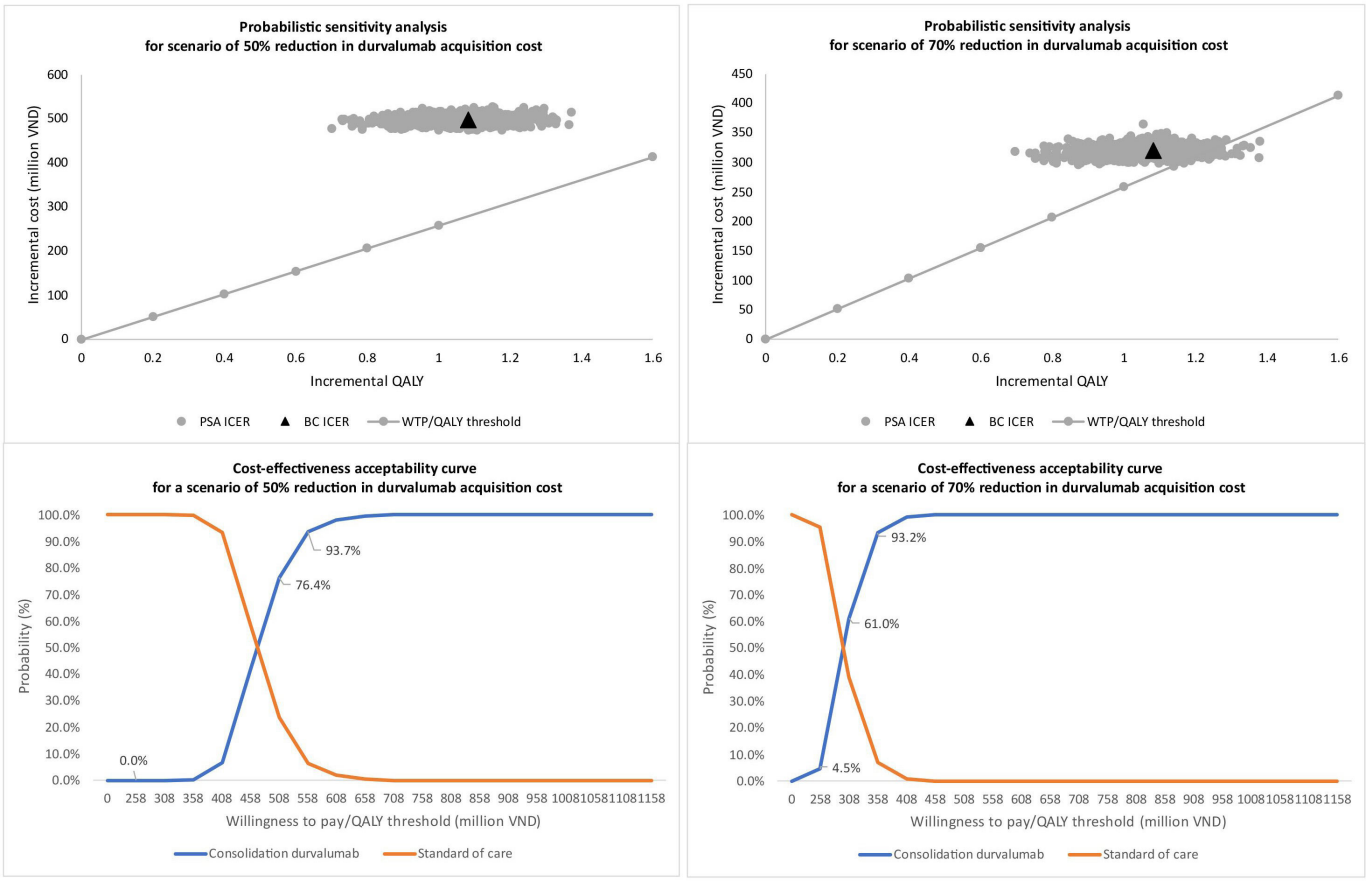
Probabilistic sensitivity analysis from a partial societal perspective.

The probability sensitivity analysis considered two scenarios: a 50% and a 70% reduction in durvalumab acquisition cost from both healthcare and partial societal perspectives. When the cost was reduced by 50%, durvalumab consolidation was not cost-effective from either perspective (figures2  3). In this scenario, the probability of cost-effectiveness exceeded 70% at thresholds of 458 million and 508 million VND per QALY gained from healthcare and partial societal views, respectively (figures2  3). With a 70% cost reduction, the probability of the cost-effectiveness of the duvalumab intervention was around 6% (partial societal) and 50% (healthcare). The cost-effectiveness acceptability curve showed a probability of over 90% for cost-effectiveness at thresholds of 308 million and 358 million VND per QALY gained for healthcare and partial societal perspectives (figures2  3).

**Figure 3:**
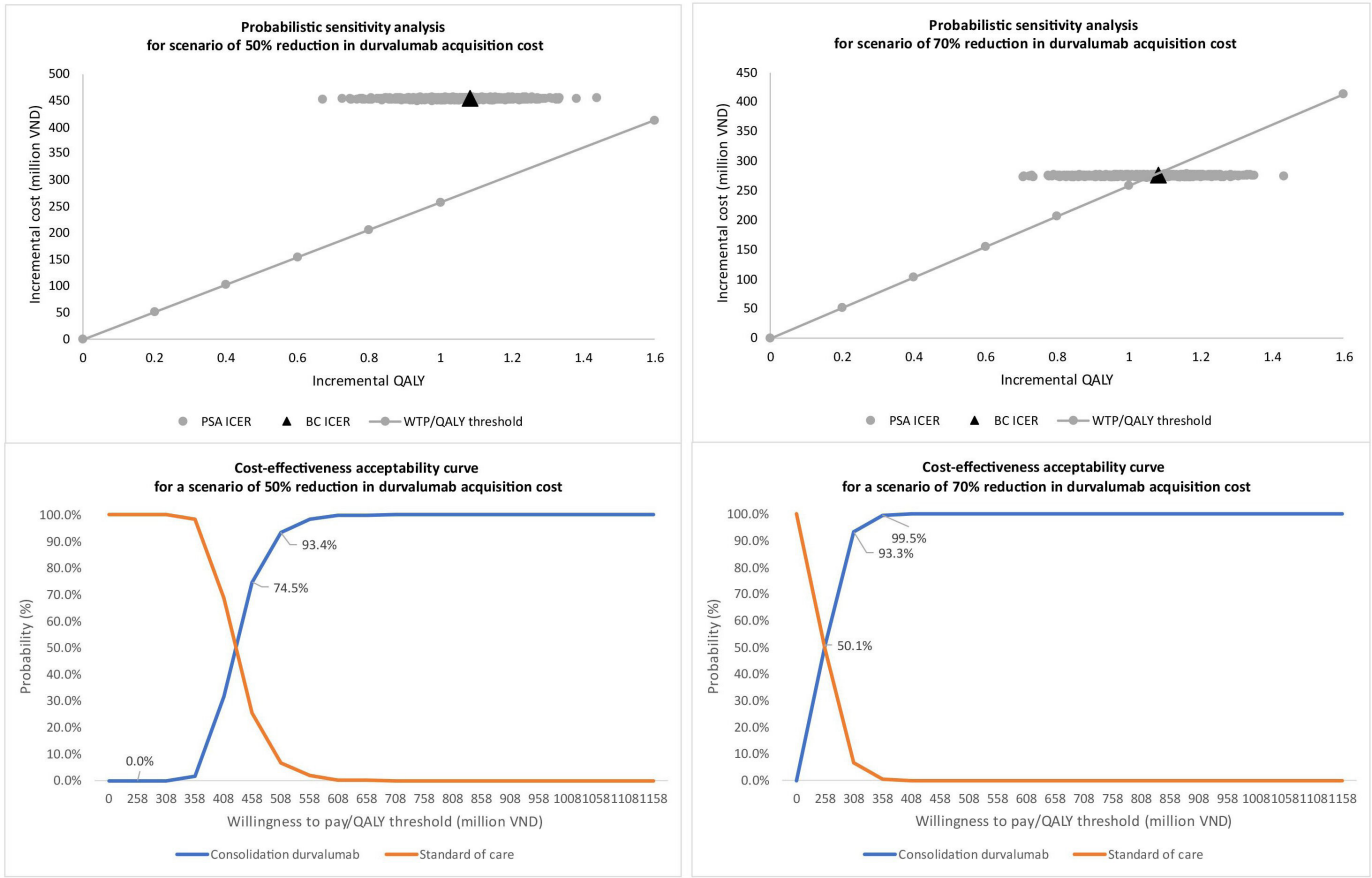
Probabilistic sensitivity analysis from a healthcare perspective.

## Discussion

This study presents local evidence of the cost-effectiveness of durvalumab for the treatment of inoperable stage III NSCLC in Vietnam. The findings indicate that the intervention is not cost-effective, even with a 50% reduction in the cost of durvalumab, from both healthcare and partial societal perspectives. However, there is a greater likelihood of cost-effectiveness if the acquisition cost of durvalumab is reduced by 70%. It is important to highlight that Vietnamese NSCLC patients experience significantly lower quality of life compared with the general population,^34^ emphasising the benefits of the consolidation durvalumab intervention in prolonging PF periods and improving the overall quality of life. Moreover, the consolidation durvalumab intervention is recommended in the national guidelines for the treatment of inoperable stage III NSCLC,^2^ indicating its acceptance among clinical experts and healthcare authorities. If durvalumab were to be included in the SHI drug list, financial support could be provided to patients, which would likely face minimal objections from clinical experts. However, it is important to consider that the inclusion of durvalumab could require trade-offs with other cost-effective drugs or services on the SHI list. Given Vietnam aims to achieve UHC by expanding SHI coverage, enhancing the package with essential drugs and services and managing the financial burden on patients, this study provides timely support for health technology assessment in the inclusion assessment of durvalumab in the SHI drug list and determining potential reimbursement rates. We suggest including durvalumab in the SHI if its acquisition cost can be reduced by 70%.

The ICER for durvalumab was found to be below the cost-effectiveness acceptability thresholds used in the USA, Switzerland and China,^14^,^18^ but above that used in Italy, a subgroup analysis in China,^18 19^ and the present study. All studies employed similar cost-effectiveness analysis methodologies, using survival data from the PACIFIC trial and varying durations (24 or 36 months) of observation and incorporating local cost and utility inputs. Our study is the first published study to consider patients’ out-of-pocket expenditure and used 5 years of survival data, thus constituting a valuable addition to existing knowledge.^35^ The cost per QALY gained was lower in Vietnam (equivalent to 37 000 USD) than in other countries (ranging from 24 397 USD in China to more than 137 000 USD in the USA). All of the mentioned studies used GDP-based acceptability thresholds, and the intervention was deemed to be not cost-effective in most scenarios in studies conducted in Italy and China and in our study. The US studies used thresholds that ranked the cost-effectiveness of an intervention highly if the ICER was lower than 50 000 USD/QALY and at an intermediate level if the ICER fell between 50 000 and 150 000 USD/QALY. However, it is important to acknowledge that the USA GDP-based threshold is sometimes perceived as higher than that of other countries.^36^ The variation in GDP-based cost-effectiveness threshold in each country significantly affects the conclusions presented here.

It is worth noting that the sustainability of such a substantial discount on the acquisition cost as is posited herein may raise concerns. Furthermore, the conclusion regarding the cost-effectiveness of the consolidation durvalumab intervention varied depending on the perspective: from a healthcare perspective, the intervention was considered cost-effective when the drug acquisition cost was reduced by 70% but from a partial societal perspective did not meet the cost-effectiveness criteria. The healthcare perspective is commonly employed as it aligns well with the objectives of policymakers when making decisions regarding resource allocation. It primarily focuses on direct medical costs, including medication expenses, administration, monitoring, adverse-event management and other medical services associated with disease management. The societal perspective, in contrast, takes a broader view by incorporating healthcare costs, out-of-pocket expenses on the part of patients and productivity loss due to disease and can reflect all aspects related to the disease. The healthcare perspective helps mitigate bias against individuals who are not actively employed, such as those of retirement age or individuals unable to work. Overall, there is no ‘gold standard’ for determining the best perspective to approach in HTA. Therefore, we have presented both the healthcare and partial societal perspectives to address this issue.

Along with the common approach of decision-making based on value for money (using a measure such as ICER) among HTA bodies, the resource-allocation process often follows ethical principles such as the need, intervention prognosis and equal claims.^37 38^ The Vietnamese Ministry of Health follows such principles in making decisions on which medicines are to be included in the SHI. Hence, evidence on the cost-effectiveness of an intervention is just one element of the decision-making process as a whole, and cost-effectiveness arguments alone cannot guide decision-makers in Vietnam. Nonetheless, the evidence presented in our study can facilitate the determination of an appropriate reimbursement rate for the drug, considering various cost-effectiveness scenarios.

Several limitations of this study should be acknowledged: first, the clinical inputs, including survival data and probability of adverse events, were derived from the PACIFIC trial, which may raise concerns about their representativeness for Vietnamese inoperable stage III NSCLC patients. However, due to limited data on a national clinical level, the present study used data from the PACIFIC trial. The study is important and timely for Vietnam in the context of the country preparing to update the SHI drugs list. Moreover, borrowing clinical data from the PACIFIC trials is a common approach that has been adopted in previous studies.^14^,^19^

Second, this study does not provide evidence of cost-effectiveness from a broader societal perspective. Other considerations, such as productivity loss due to durvalumab, should be taken into consideration. In addition, we assumed that the costs for treatments related to mild and moderate adverse events would not affect the cost-effectiveness model; hence, they were not included in our estimates. Additionally, the cost of terminal care was excluded due to data limitations. This is a limitation of our study, as we could not account for all potential costs arising from adverse events or terminal care. Furthermore, various methods exist for estimating cost in economic models, such as the claim-based, microcosting and guideline-based methods (the latter of which was used in the present study). Each method can yield significantly different results and lead to a different conclusion regarding the cost-effectiveness of an intervention. Due to current policy restrictions set by the SHI, we were unable to access the SHI’s claim-based system. While the system’s management unit shared a lump-sum figure for the total annual reimbursement for drugs and services, they did not provide details regarding the cost breakdown for drugs and services based on disease and disease stages. Microcosting was considered impractical due to the extensive resource requirements in terms of both finances and time. Consequently, we opted to employ the guideline-based method for cost estimation in this study. This relied on the SHI’s reimbursement prices, taking into account their estimations of the necessary resources for personnel, drugs, medical materials and equipment. However, it remains uncertain whether these estimations encompass other recurring costs, such as training, operation, maintenance and associated capital costs (eg, depreciation of buildings and equipment). Nonetheless, we made every effort to collect all available local data, and this limitation highlights the need for further research when more comprehensive data becomes available.

Third, this study used a partitioned survival model exclusively for the development of the cost-effectiveness evaluation. The decision to use a partitioned survival model was based on two factors^1^: the model directly incorporated clinical trial endpoints as stage probabilities without necessitating individual data assessment, and^2^ individual data, either from PACIFIC trial or a national database, was not available. However, existing literature suggests that survival extrapolations derived from a partitioned survival model may differ significantly from those obtained using a regular stage transitional model, such as the Markov model.^39^ Consequently, both the stage transitional model and the partitioned survival model should complement each other in assessing clinical uncertainties during the extrapolation period.^39^ It is recommended that future cost-effectiveness studies use both a stage transitional model and a partitioned survival model when individual clinical data can be accessed.

Fourth, the local data assumed that costs and utilities associated with the disease stages IIIB and IV were similar to those observed in the model stages (PF and PD). There is a possibility that patients may progress while remaining in the same disease stage, introducing ambiguity in the model estimation. However, given the scarcity of information, we leveraged all available local data in our cost-effectiveness analysis. Additionally, sensitivity analysis was performed to mitigate uncertainties associated with the inputs used.

Finally, the evaluation of the cost-effectiveness of the intervention was conducted using a proposed GDP-based threshold, which is an approach that has received significant criticism.^40^ Concerns have been raised about the relevance of the thresholds to the country’s health budget, the reliability of GDP-based thresholds themselves and the possibility of them inadequately appraising affordability.^40^ To address any uncertainty stemming from the cost-effectiveness threshold, we conducted cost-effectiveness acceptability curve analyses to examine the sensitivity of the ICER/NMB at various thresholds. In other words, we provided the Ministry of Health with references regarding the probabilities of cost-effectiveness for consolidation durvalumab in inoperable stage III NSCLC at different levels of their accepted thresholds.

## Conclusion

This study provides local evidence regarding the cost-effectiveness of durvalumab for the treatment of inoperable stage III NSCLC in various scenarios in Vietnam. The intervention was found to be not cost-effective at full acquisition cost, but it is important to acknowledge that cost-effectiveness arguments alone cannot guide decision-makers in Vietnam; other criteria, such as budget impact and ethical concerns, are crucial factors to consider within decision-making processes.

## supplementary material

10.1136/bmjopen-2024-083895 online supplemental file 1

10.1136/bmjopen-2024-083895 online supplemental file 2

10.1136/bmjopen-2024-083895 online supplemental file 3

## Data Availability

All data relevant to the study are included in the article or uploaded as supplementary information.
