# Supplementary material for: Cost-effectiveness of consolidation durvalumab for inoperable stage III non-small cell lung cancer in Vietnam
Source: BMJ Open. 2024 Aug 30;14(8):e083895. doi: 10.1136/bmjopen-2024-083895 (PMC11407225; doi:10.1136/bmjopen-2024-083895)
Supplement: online supplemental file 1 [file bmjopen-14-8-s001.pdf]

Supplemental Figure 1. Non-small cell lung cancer treatment in Vietnam.

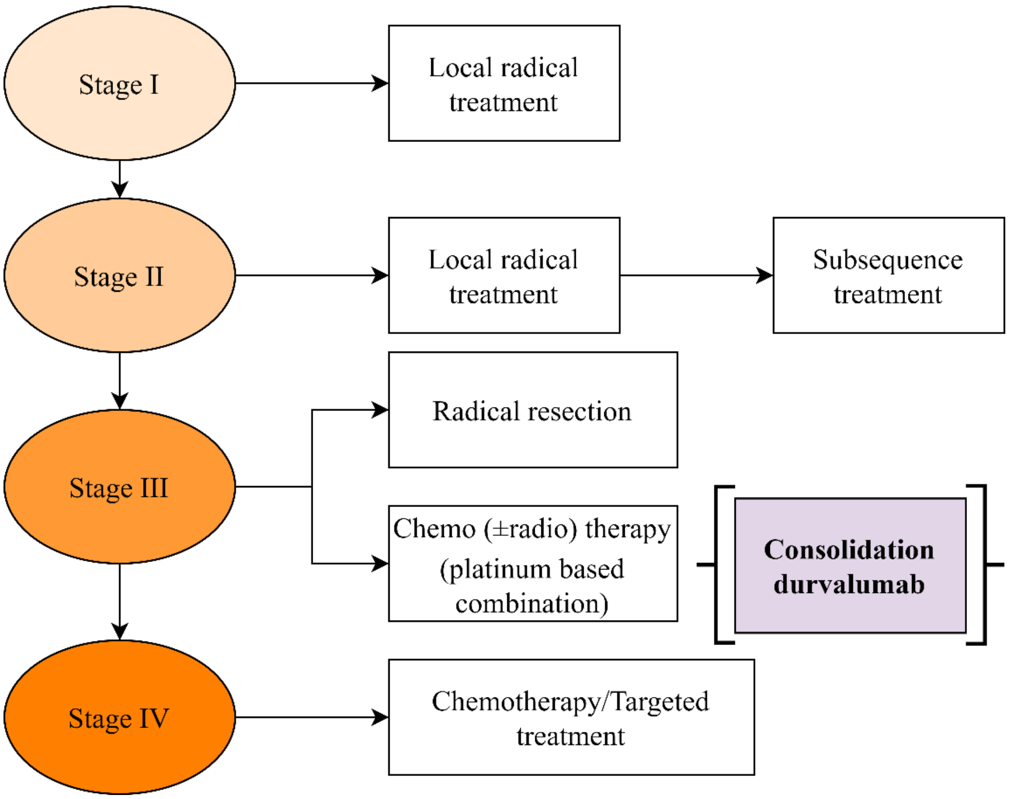

Supplemental Figure 2. The partitioned survival model for cost-effectiveness analysis.

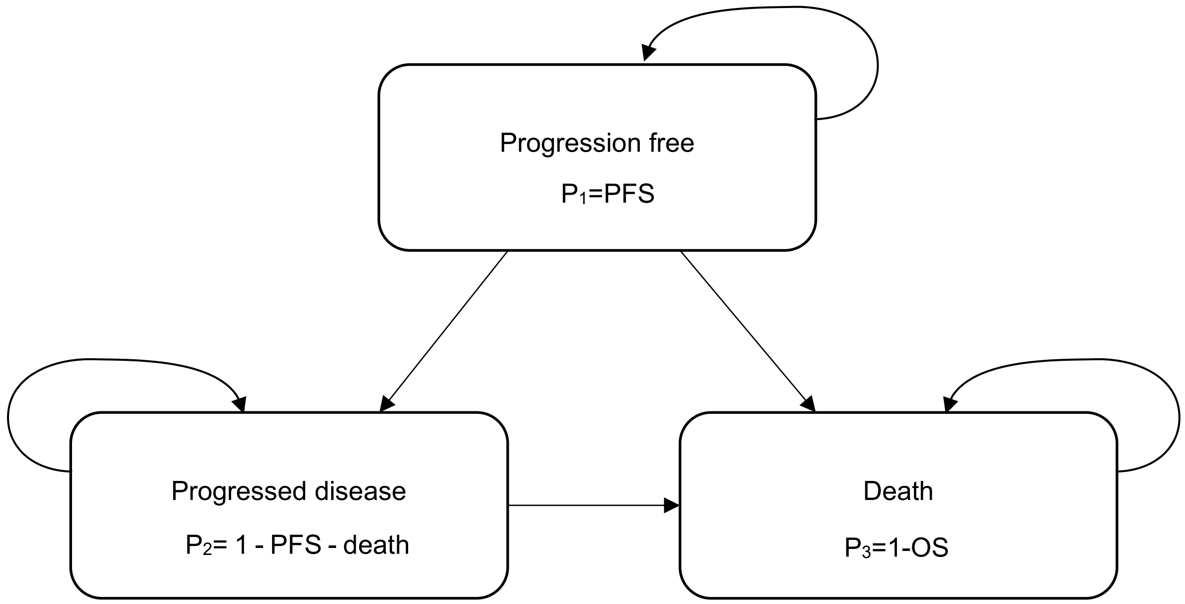

Notes: P1-3: Probability at each stage. PFS: progression-free survival rate. OS: overall survival rate.

Supplemental Figure 3. Overall survival rate and progression-free survival rate used in the cost effectiveness model.

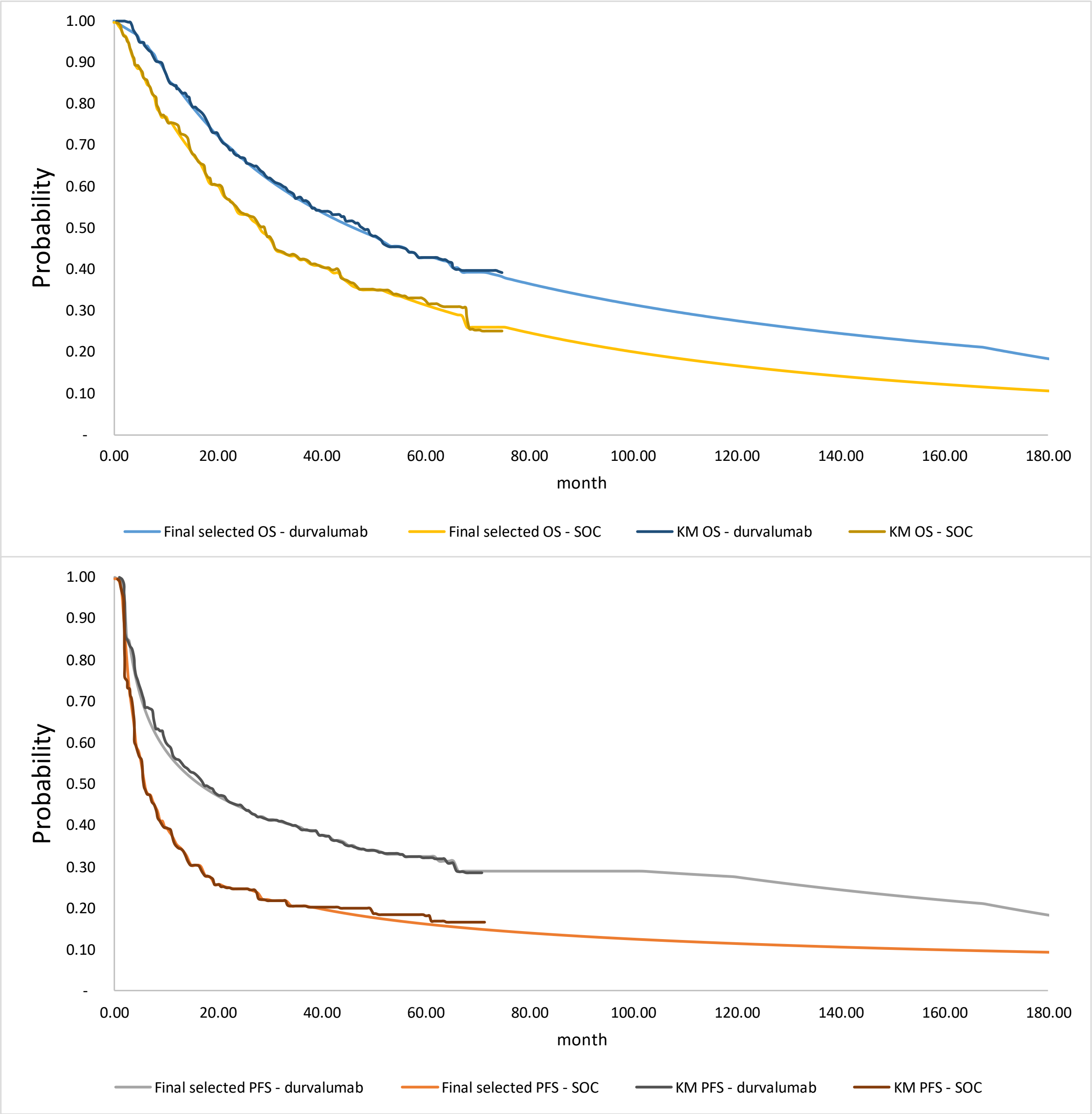

PFS: progression-free survival. OS: overall survival. KM: Kaplan Meier
