# Supplementary material for: Cost-effectiveness of consolidation durvalumab for inoperable stage III non-small cell lung cancer in Vietnam
Source: BMJ Open. 2024 Aug 30;14(8):e083895. doi: 10.1136/bmjopen-2024-083895 (PMC11407225; doi:10.1136/bmjopen-2024-083895)
Supplement: Supplementary file 4 [file bmjopen-2024-083895.reviewer_comments.pdf]

PEER REVIEW HISTORY

BMJ Open publishes all reviews undertaken for accepted manuscripts. Reviewers are asked to complete a checklist review form and are provided with free text boxes to elaborate on their assessment. These free text comments are reproduced below.

ARTICLE DETAILS

**TITLE (PROVISIONAL)**	Cost-effectiveness of consolidation durvalumab for inoperable stage III non-small cell lung cancer in Vietnam
**AUTHORS**	Vu, Mai; Lindholm, Lars; Van Minh, Hoang; Sun, Sun; Kim, Giang; Sahlén, Klas-Göran

VERSION 1 - REVIEW

**REVIEWER NAME**	Baldo, Vincenzo
**REVIEWER AFFILIATION**	University of Padua
**REVIEWER CONFLICT OF INTEREST**	none
**DATE REVIEW RETURNED**	03-Apr-2024

**GENERAL COMMENTS**	The present work aimed to assess the cost-effectiveness of durvalumab for patients with inoperable stage III non-small cell lung cancer from healthcare and partial societal perspectives in Vietnam. The work has special value for Vietnam where durvalumab is suggested in the national treatment guidelines, but it is not covered by national health insurance, hence pushing cancer patients into poverty risk.<br>The rationale, the methodology and the data sources are rigorously detailed. The results and the study limitation are well discussed. Only few minor comments follow.<br><br>Minor comments:<br>• Pag 6, Lines 16-18: “This inoperable stage III NSCLC population is typically managed through concurrent or sequential chemoradiotherapy (2), the five-year overall survival rate for which is unfortunately below 25% (4)”. The sentence seems a bit ambiguous, I would suggest rephrasing it.<br>• Pag 6, Line 38: “and anaemia (7).According to the pharmaceutical provider”. Add space after (7).<br>• Pag 7, Lines 4-6: “Durvalumab, a salvage treatment for inoperable stage III NSCLC, is currently not included in the SHI scheme”. This sentence is a repetition of what already stated only few lines above “durvalumab […] it is not covered by national health insurance in Vietnam”. I would suggest rephrasing it.<br>• Pag 9, Lines 24-28: “In the present study, the lead author conducted additional analysis on patient’s utility at different disease stages using the anonymised dataset provided from Ha, Hoang (20)’s study. This analysis involved data from 400 NSCLC adult”. Could you please briefly expand this paragraph by better explaining what methods you used in this utility analysis and the rationale?<br>• Pag 10, Lines 38-41: “The cost of adverse-event treatments (Component 5) consisted of the expenses associated with managing four severe adverse events: pneumonia, pneumonitis, anaemia, and dyspnea”. Please include in the study limitation paragraph that other adverse event costs are not accounted for. Additionally, consider

	discussing the common risk/practice in cost-effectiveness analysis studies of omitting indirect costs, potentially leading to an underestimation of overall costs.<br>• Several studies (like Laudicella M, Walsh B, Burns E, Smith PC. Cost of care for cancer patients in England: evidence from population-based patient-level data. Br J Cancer. 2016 May 24;114(11):1286-92. doi: 10.1038/bjc.2016.77) reported that the highest average costs are observed in the months immediately preceding death (the 'terminal' phase). Is this finding consistent with the expenses you associated to the PD or D states of your model?<br>• Figure 2. The labels on the CE acceptability curve x-axis are not easy to read (after 1,000 VND). Please fix them.

**REVIEWER NAME**	Wei, Shuang
**REVIEWER AFFILIATION**	National Health Commission (NHC) Key Laboratory of Respiratory Disease, Tongji Hospital
**REVIEWER CONFLICT OF INTEREST**	There is no conflict of interests.
**DATE REVIEW RETURNED**	29-Apr-2024

**GENERAL COMMENTS**	This study assessed the cost-effectiveness of using durvalumab for the treatment of inoperable stage III non-small cell lung cancer in Vietnam using a lifetime partitioned survival model, which may provide guidance for clinical decision-making. However, in the discussion section, the authors should compare the results of the study with related studies in other countries or regions, which would help to reveal possible differences in the cost-effectiveness of the drug under different healthcare systems and provide a broader perspective on healthcare decision-making on a global scale.

VERSION 1 – AUTHOR RESPONSE

Reviewer: 1. Dr. Vincenzo Baldo, University of Padua

Comments to the Author:

The present work aimed to assess the cost-effectiveness of durvalumab for patients with inoperable stage III non-small cell lung cancer from healthcare and partial societal perspectives in Vietnam. The work has special value for Vietnam where durvalumab is suggested in the national treatment guidelines, but it is not covered by national health insurance, hence pushing cancer patients into poverty risk. The rationale, the methodology and the data sources are rigorously detailed. The results and the study limitation are well discussed. Only few minor comments follow.

Minor comments:

Pag 6, Lines 16-18: "This inoperable stage III NSCLC population is typically managed through concurrent or sequential chemoradiotherapy (2), the five-year overall survival rate for which is unfortunately below 25% (4)". The sentence seems a bit ambiguous, I would suggest rephrasing it.

Response: Thank you for your comment. We revised the sentence as below:

"Patients with inoperable stage III NSCLC are typically managed through concurrent or sequential chemoradiotherapy (2). However, evidence indicates that the five-year overall survival rate for these patients is unfortunately below 25% (4)."

Pag 6, Line 38: "and anaemia (7).According to the pharmaceutical provider". Add space after (7).

Response: Thank you for your comment. We revised accordingly.

Pag 7, Lines 4-6: "Durvalumab, a salvage treatment for inoperable stage III NSCLC, is currently not included in the SHI scheme". This sentence is a repetition of what already stated only few lines above "durvalumab […] it is not covered by national health insurance in Vietnam". I would suggest rephrasing it.

Response: Thank you for your comment. We revised the sentence as below:

"Durvalumab is currently not included in the SHI scheme, meaning that patients hoping to extend their lives with this immunotherapy must bear the entire cost themselves, causing a substantial financial burden for them and their families."

Pag 9, Lines 24-28: "In the present study, the lead author conducted additional analysis on patient's utility at different disease stages using the anonymised dataset provided from Ha, Hoang (20)'s study. This analysis involved data from 400 NSCLC adult". Could you please briefly expand this paragraph by better explaining what methods you used in this utility analysis and the rationale?

Response: Thank you for your comment. In fact, the utility presented in the article by Ha, Hoang (20) had been calculated using an informal EQ-5D-5L value set and they published only the mean value of the NSCLC population, hence, in this analysis we would like to update the utility with the latest EQ-5D-5L value set and also we do the analysis to explore mean utility by disease stages.

The rationale of this additional analysis has been explained in lines 22 – 23:

"The utility of Vietnamese NSCLC patients was previously reported by Ha, Hoang (20) in 2018, where a ***mean utility of 0.66*** *was derived using the EQ-5D-5L instrument and a* ***pre-published Vietnamese value set.****"*

Hence, the method of this extra analysis has been describle in lines 30 -39:

"*The health utility values were re-calculated* ***using the recently published Vietnam EQ-5D-5L value set*** *(28). The* ***mean utility for NSCLC patients in stage IIIB*** *was found to be 0.76, while for those in* ***stage IV*** *it was 0.68 (Table 1). Due to data limitations, we assumed equivalent mean utilities for PF and stage IIIB patients and for PD and stage IV patients (Table 1)."*

Pag 10, Lines 38-41: "The cost of adverse-event treatments (Component 5) consisted of the expenses associated with managing four severe adverse events: pneumonia, pneumonitis, anaemia, and dyspnea". Please include in the study limitation paragraph that other adverse event costs are not accounted for. Additionally, consider discussing the common risk/practice in cost-effectiveness analysis studies of omitting indirect costs, potentially leading to an underestimation of overall costs.

Response: Thank you for your comment. We revised the discussion accordingly.

"Secondly, this study does not provide evidence of cost-effectiveness from a broader societal perspective. Other considerations, such as productivity loss due to durvalumab, should be taken into consideration. In addition, we assumed that the costs for treatments related to mild and moderate adverse events would not affect the cost-effectiveness model; hence, they were not included in our estimates. Additionally, the cost of terminal care was excluded due to data limitations. This is a limitation of our study, as we could not account for all potential costs arising from adverse events or terminal care."

Several studies (like Laudicella M, Walsh B, Burns E, Smith PC. Cost of care for cancer patients in England: evidence from population-based patient-level data. Br J Cancer. 2016 May 24;114(11):1286-92. doi: 10.1038/bjc.2016.77) reported that the highest average costs are observed in the months immediately preceding death (the 'terminal' phase). Is this finding consistent with the expenses you associated to the PD or D states of your model?

Response: Thank you for your comment. You are correct. However, terminal care is not standardized in Vietnam and is also not covered by health insurance, making it difficult for us to estimate terminal care costs in our study. Some aspects of terminal care may be included in out-of-pocket (OOP) payments, but we are not certain because OOP costs were captured through patient interviews, making it difficult to stratify the amount specifically for terminal care. Hence, we acknowledge this as one of our study's limitations, please check the text at above comment.

Figure 2. The labels on the CE acceptability curve x-axis are not easy to read (after 1,000 VND). Please fix them.

Response: Thank you for your comment. We tried to revise it; however, adjusting the axis is quite difficult because we have to combine many elements into one figure. Since the CE acceptability curve has been stable after around 400-700 million dong, the figure beyond 1000 may not be necessarily important.

Reviewer: 2. Dr. Shuang Wei, National Health Commission (NHC) Key Laboratory of Respiratory Disease, Tongji Hospital. Comments to the Author:

This study assessed the cost-effectiveness of using durvalumab for the treatment of inoperable stage III non-small cell lung cancer in Vietnam using a lifetime partitioned survival model, which may provide guidance for clinical decision-making. However, in the discussion section, the authors should compare the results of the study with related studies in other countries or regions, which would help to reveal possible differences in the cost-effectiveness of the drug under different healthcare systems and provide a broader perspective on healthcare decision-making on a global scale.

Response: Thank you for your comment. In our discussion, we compared our ICER from the presented study with results from published studies conducted in the USA, Switzerland, China, and Italy. We observed significant differences across countries, primarily because each study used local sources to estimate costs. To discuss these variations, we focused on comparing the methods used and the CEA thresholds applied in each study, which provided a clearer understanding of the ICER differences. This part of the discussion is pasted here for your consideration.

"The ICER for durvalumab was found to be below the cost-effectiveness acceptability thresholds used in the USA, Switzerland, and China (14-18), but above that used in Italy, a subgroup analysis in China, (18, 19), and the present study. All studies employed similar cost-effectiveness analysis methodologies, utilising survival data from the PACIFIC trial and varying durations (24 or 36 months) of observation and incorporating local cost and utility inputs. Our study is the first published study to consider patients' out-of-pocket expenditure and utilised five years of survival data, thus constituting a valuable addition to existing knowledge (35). The cost per QALY gained was lower in Vietnam (equivalent to 37,000 USD) than in other countries (ranging from 24,397 USD in China to more than 137,000 USD in the USA). All of the mentioned studies utilised GDP-based acceptability thresholds, and the intervention was deemed to be not cost-effective in most scenarios in the studies conducted in Italy and China and in our study. The USA studies used thresholds that ranked the cost-effectiveness of an intervention highly if the ICER was lower than 50,000 USD/QALY, and at an intermediate level if the ICER fell between 50,000 and 150,000 USD/QALY. However, it is important to acknowledge that the USA GDP-based threshold is sometimes perceived as higher than that of other countries (36). The variation in GDP-based cost-effectiveness threshold in each country significantly affects the conclusions presented here."
